# Two cases demonstrate an association between *Tropheryma whipplei* and pulmonary marginal zone lymphoma

**DOI:** 10.1186/s13027-024-00597-0

**Published:** 2024-07-27

**Authors:** J. D. Haslbauer, C. Wiegand, B. Hamelin, V. S. Ivanova, T. Menter, S. Savic Prince, A. Tzankov, K. D. Mertz

**Affiliations:** 1 Institute of Medical Genetics and Pathology, University Hospital Basel, Basel, Switzerland; 2 Institute of Pathology, Cantonal Hospital Baselland, Mühlemattstrasse 11, CH-4410 Liestal, Switzerland; 3 University of Basel, Basel, Switzerland

**Keywords:** Pulmonary marginal zone lymphoma, MALT lymphoma, *Tropheryma whipplei*, *Achromobacter xylosoxidans*, Whipple’s disease, Pulmonary microenvironment, Metagenomics whole genome sequencing, Antibiotics

## Abstract

**Background:**

Marginal zone lymphomas of mucosa-associated lymphatic tissues (MZL of MALT) are a group of indolent B-cell neoplasms, which are thought to arise from chronic antigenic stimulation of B-cells either due to underlying chronic infection or autoimmune disease. Little is known about potential causative pathogens in pulmonary MZL (PMZL), although some data suggests a potential role of *Achromobacter* (*A.*) *xylosoxidans*.

**Methods:**

An index case of chronic pulmonary colonisation with *Tropheryma* (*T.*) *whipplei* and subsequent development of PMZL was identified by *T. whipplei* specific PCR and metagenomic next genome sequencing (mNGS). This case prompted a retrospectively conducted analysis of *T. whipplei-*specific PCRs in lung tissue from PMZL patients (n = 22), other pulmonary lymphomas, and normal controls. Positive results were confirmed by mNGS. A systematic search for *T. whipplei* and *A. xylosoxidans* in our in-house mNGS dataset comprising autopsy lungs, lung biopsies and lung resection specimens (n = 181) was subsequently performed.

**Results:**

A 69-year-old patient presented with weight loss and persistent pulmonary consolidation. Subsequent mNGS analysis detected *T. whipplei* in the resected lung specimen. An antibiotic regimen eventually eliminated the bacterium. However, the consolidation persisted, and the diagnosis of PMZL was made in a second lung resection specimen. A second case of *T. whipplei*-associated PMZL was subsequently detected in the retrospectively analysed PMZL cohort. Both cases showed comparatively few mutations and no mutations in genes encoding for NF-κB pathway components, suggesting that *T. whipplei* infection may substitute for mutations in these PMZL. None of the samples in our in-house dataset tested positive for *T. whipplei*. In contrast, *A. xylosoxidans* was frequently found in both autopsy lungs and lung biopsy / resection specimens that were not affected by PMZL (> 50%).

**Conclusions:**

Our data suggests that *T. whipplei* colonisation of lungs may trigger PMZL as a potential driver. Systematic analyses with larger cohorts should be conducted to further support this hypothesis. The frequent detection of *A. xylosoxidans* in lung tissue suggests that it is a common component of the pulmonary microbiome and therefore less likely to trigger lymphomas.

**Supplementary Information:**

The online version contains supplementary material available at 10.1186/s13027-024-00597-0.

## Background

In 2018, 2.2 million infection-attributable cancer cases were diagnosed worldwide [1]. To optimise screening programs and antineoplastic therapy in infection-associated subtypes of cancer, it is thus necessary to study the crosstalk of infections and neoplasia. While carcinogenesis caused by viruses such as human papillomavirus (HPV) and Epstein-Barr virus (EBV) is extensively explored, comparatively less is known about the relationship between certain bacterial infections and neoplasia.

Marginal zone lymphomas of mucosa-associated lymphatic tissues (MZL of MALT) are a group of indolent B-cell neoplasms that are thought to arise from chronic antigenic stimulation in mucous membranes, either due to an infectious agent or underlying autoimmune disease [2–4]. Certain bacteria are known or suspected to be associated with MZL of MALT lymphomagenesis, e.g. *Helicobacter pylori* in gastric MZL [5], *Chlamydia psittaci* in ocular adnexal MZL [6], and *Borrelia burgdorferi* in cutaneous MZL [7]. The lung parenchyma hosts a dynamic and heterogeneous microbiome, which makes it an interesting source of study for potential lymphomagenic infections [8]. Microbiological studies suggested a potential role of *Achromobacter* (*A.*) *xylosoxidans* in the pathogenesis of pulmonary MZL (PMZL) [9], which was subsequently challenged by metagenomics analyses [10]. Therefore, systematic research is required to identify potential infectious agents which may play a causative role in the pathogenesis of PMZL.

*Tropheryma* (*T.*) *whipplei*, a Gram-positive rod-shaped bacterium of the phylum *Actinomycetota*, is the causative organism of Whipple’s disease, a systemic disorder affecting the gastrointestinal tract and other organ systems including the cardiovascular system, central nervous system, and joints. The only known reservoir of *T. whipplei* in humans is the gastrointestinal tract, where the bacteria accumulate in macrophages of the lamina propria [11]. Chronic carrier rate in the population is high (up to 20% in specific populations such as sewage workers, HIV-infected and the homeless [11–13]), while the development of the full clinical picture of Whipple’s disease is markedly rare (incidence < 1/1′000′000). This suggests that potential hereditary [14], gender-specific and/or environmental factors [12] additionally play a role in developing symptomatic disease. Typical Whipple’s disease patients are middle-aged Caucasian males who present with diarrhoea, arthralgia, and fever. Chronic disease can manifest in different ways, ranging from endocarditis, uveitis, polyarthritis to encephalitis [15, 16]. Pulmonary infections are rare, though cases of both acute and chronic pneumonia, interstitial lung disease, and pulmonary hypertension in association with *T. whipplei* have been reported [15, 17]. Isolated reports in the literature have described cases with chronic *T. whipplei* colonisation associated with B-cell neoplasia [18–20].

We report a case of a 69-year-old female with chronic pulmonary Whipple’s disease who subsequently developed PMZL. This case prompted us to systematically analyse a series of PMZL and other pulmonary lymphomas by means of *T. whipplei* PCR and metagenomic next genome sequencing (mNGS) to explore a potential pathophysiological link between *T. whipplei* and PMZL. In this analysis, we found a second case of *T. whipplei*-positive PMZL. This suggests that chronic *T. whipplei* infections may be associated with PMZL, may even promote lymphomagenesis, and/or that neoplasias such as PMZL may provide a niche for *T. whipplei* persistence.

## Materials and methods

### Patient selection

Lung tissue containing PMZL (n = 22), other pulmonary lymphomas (n = 8), and normal lung tissue (n = 3) was retrieved from the archives of the Institutes of Pathology of the University Hospital Basel and of the Cantonal Hospital Baselland, Switzerland (2005–2022) [21]. Clinical data collected for each case include age, sex, date of diagnosis, follow-up, and history of antibiotic intake. At least one representative sample of lung tissue containing PMZL was examined. Due to a potential heterogeneity of microbial load, two or three lung samples from varying anatomical locations were analysed in seven cases (Table 1).

**Table 1:** Whipple PCRs on a cohort of 22 PZML. In cases with sufficient clinical data, a minimum 7-day intake of antibiotics within a period of 4 weeks prior to tissue sampling was documented

Patient	Sex	Age	Sample	*T. whipplei* PCR	Antibiotic intake
1	Male	57	Left upper lobe	Negative	Yes: Cefazolin (2 g)
			Left upper lobe	Negative	Yes: Cefazolin (2 g)
			Right upper lobe	Negative	Yes: Cefazolin (4 g)
2	Male	36	Lower lobe	Positive	None
			Lower lobe	Positive	None
3	Male	48	Left lower lobe	Negative	None
			Left lower lobe	Negative	None
4	Female	74	Right upper lobe	Negative	Yes: Cefuroxim (1.5 g)
			Apical upper lobe	Negative	Yes: Cefazolin (2 g)
			Apical lower lobe	Negative	Yes: Cefazolin (2 g)
5	Male	79	Left upper lobe	Negative	None
6	Female	61	Left lower lobe	Negative	No information
7	Male	71	Right lower lobe	Negative	None
8	Male	46	Right lower lobe	Negative	Yes: Amoxicillin, Clavulanate
			Right lower lobe	Negative	Yes: Amoxicillin, Clavulanate
9	Male	71	Right lower lobe	Negative	No information
10	Female	76	Right lower lobe	Negative	No information
			Right lower lobe	Negative	No information
11	Male	72	Right lower lobe	Negative	Yes: Amoxicillin, Clavulanate
12	Male	80	Right middle lobe	Negative	None
13	Female	64	Left upper lobe	Negative	No information
14	Female	71	Right lower lobe	Negative	None
15	Male	67	Left lower lobe	Negative	None
16	Female	50	Right middle lobe	Negative	None
17	Male	17	Left upper lobe	Negative	No information
18	Male	77	Left lower lobe	Negative	No information
19	Female	67	Left upper lobe	Negative	No information
20	Male	60	Left upper lobe	Negative	No information
21	Female	55	Right lower lobe	Negative	No information
22	Male	81	Left lower lobe	Negative	No information

### Immunohistochemistry

Immunohistochemical double stains for PAX5 (rabbit anti-human PAX5, clone SP34, 312R, dilution 1:25, incubation 15 min; Cellmarque, Sigma-Aldrich, Rocklin, CA, USA; Bond Polymer Refine Detection DS9800), and the pan-cytokeratin AE1/AE3 (mouse anti-human cytokeratin, clone AE1/AE3, M3515, dilution 1:100, incubation 15 min; Agilent, Santa Clara, CA, USA; Bond Polymer Refine Red Detection DS9390) were performed on a Leica Bond III autostainer using EDTA buffer (pH9) for antigen retrieval (H2(30)95, AR9940) and the REFINE polymer detection system (Leica Biosystems, Newcastle, UK).

Immunohistochemistry for CD1a (mouse anti-human CD1a, clone 010, M3571, dilution 1:100, incubation 20 min; Agilent, Santa Clara, CA, USA; Bond Polymer Refine Red Detection DS9390) was also performed on a Leica Bond III autostainer using EDTA buffer (pH9) for antigen retrieval (H2(20)100, AR9940) and the REFINE polymer detection system (Leica Biosystems, Newcastle, UK).

### Nucleic acid extraction

For PCR and mNGS analyses, ten formalin-fixed, paraffin-embedded (FFPE) tissue sections were incubated with proteinase K overnight. DNA was extracted on a Qiagen EZ2 Connect device with the Qiagen EZ1&2 DNA Tissue kit (Qiagen, Hilden, Germany).

For high-throughput sequencing (HTS), DNA extraction from 10 µm thick untreated FFPE tissue was performed using the Invitrogen™ RecoverAll™ Multi-Sample RNA/DNA Workflow (#A26069, Thermo Fisher Scientific, Waltham, MA, USA).

### Polymerase chain reactions

PCR was performed with the Ampli-Taq Gold polymerase (Thermo Fisher Scientific, Waltham, MA, USA) with two sets of primers specifically targeting the 16 s and 23 s rRNA of *T. whipplei* (16 s rRNA primers: 5’-AGAGATACGCCCCCCGCAA-3’ and 5’-TCCTGTGAGTCCCCGCCATTACGC-3’ with an annealing temperature of 55 °C; 23 s rRNA primers: 5’-GGGTAGTGGGATCCTCTATGTGG-3’ and 5’-CGGGACTATCACCCTCTTCG-3’ with an annealing temperature of 55 °C). PCR products (with expected sizes of 141 and 101 base pairs, respectively) were resolved using a Qiagen Qiaxcel Connect capillary electrophoresis device.

The presence of *Mycobacterium tuberculosis* and *Mycobacterium species* (including *M. marinum, leprae, kansasii, lufu* and *haemophilum*) was assessed by a nested PCR targeting the IS6110 and a quantitative PCR targeting additional sequences (see Supplementary Table 1 for primer and probe sequences).

The presence of *Francisella tularensis* was determined via a semi-nested PCR using the following primers: primer 1–5’-TGCTGCTGCTCAGACAGCTACTA-3’, primer 2–5’-ACCTTCTGGAGCCTGCCATTGT-3’, primer 3–5’-GCAAGCTGCTGCTGTATCTAAGCCA-3’. An annealing temperature of 64 °C and the GeneAmp Fast PCR Master Mix (Thermo Fisher Scientific, Waltham, MA, USA) were used.

All PCR performed included a synthetic positive, negative, and extraction control (a kidney tissue FFPE block negative for *T. whipplei* and with good DNA quality). DNA quality was assessed in each sample via a human β-Globin PCR.

### Metagenomic next-generation sequencing (mNGS)

Shotgun metagenomic sequencing was used to sequence the microbial content of the samples without prior selection nor enrichment. NGS libraries using 250 ng of DNA from each sample were generated using the Ion Plus Fragment Library kit (Thermo Fisher Scientific, Waltham, MA, USA). Libraries were quantified using the Ion Library TaqMan Quantification kit (Thermo Fisher Scientific, Waltham, MA, USA). Sequencing was performed on an Ion Torrent GeneStudio S5XL (Thermo Fisher Scientific, Waltham, MA, USA).

### Pathogen detection by mNGS

The shotgun sequencing data was analysed with the CLC Genomics Workbench software and its Microbial Genomics module (Qiagen, Hilden, Germany) to perform taxonomic profiling and pathogen detectionas previously described [22].

### High throughput sequencing and data analysis

Mutational analysis by HTS was performed using an IonAmpliSeq™-customised, -validated and ISO15189-accredited lymphoma panel comprising 4716 amplicons (size range: 125–175) of 172 genes (Thermo Fisher Scientific, Waltham, MA, USA) [23]. Library preparation, chip loading, variant annotation and variant classification were conducted as previously described [24].

### Data availability statement

The datasets featured in this study are accessible via the European Nucleotide Archive (ENA, https://www.ebi.ac.uk/) under the accession no. PRJEB73816.

## Results

### Index patient

A 69-year-old female (subsequently referred to as “index patient”) first presented to our outpatient clinic in August 2019 with extensive weight loss (20 kg over the course of 5 months). A CT scan revealed a consolidation in the right lower pulmonary lobe. No dyspnoea or cough was reported. The patient had stopped smoking 50 years ago and has a history of 10 pack years. Bronchoalveolar lavage (BAL) and transbronchial biopsy yielded no evidence of malignancy. Cytologic examination of bronchial secretion and BAL showed mixed inflammation with a predominance of neutrophilic granulocytes, while in bronchial fluid, granulomatous inflammation was described. However, PCR analyses for *Mycobacterium spp*. and *Francisella tularensis* yielded negative results.

**Fig. 1:**
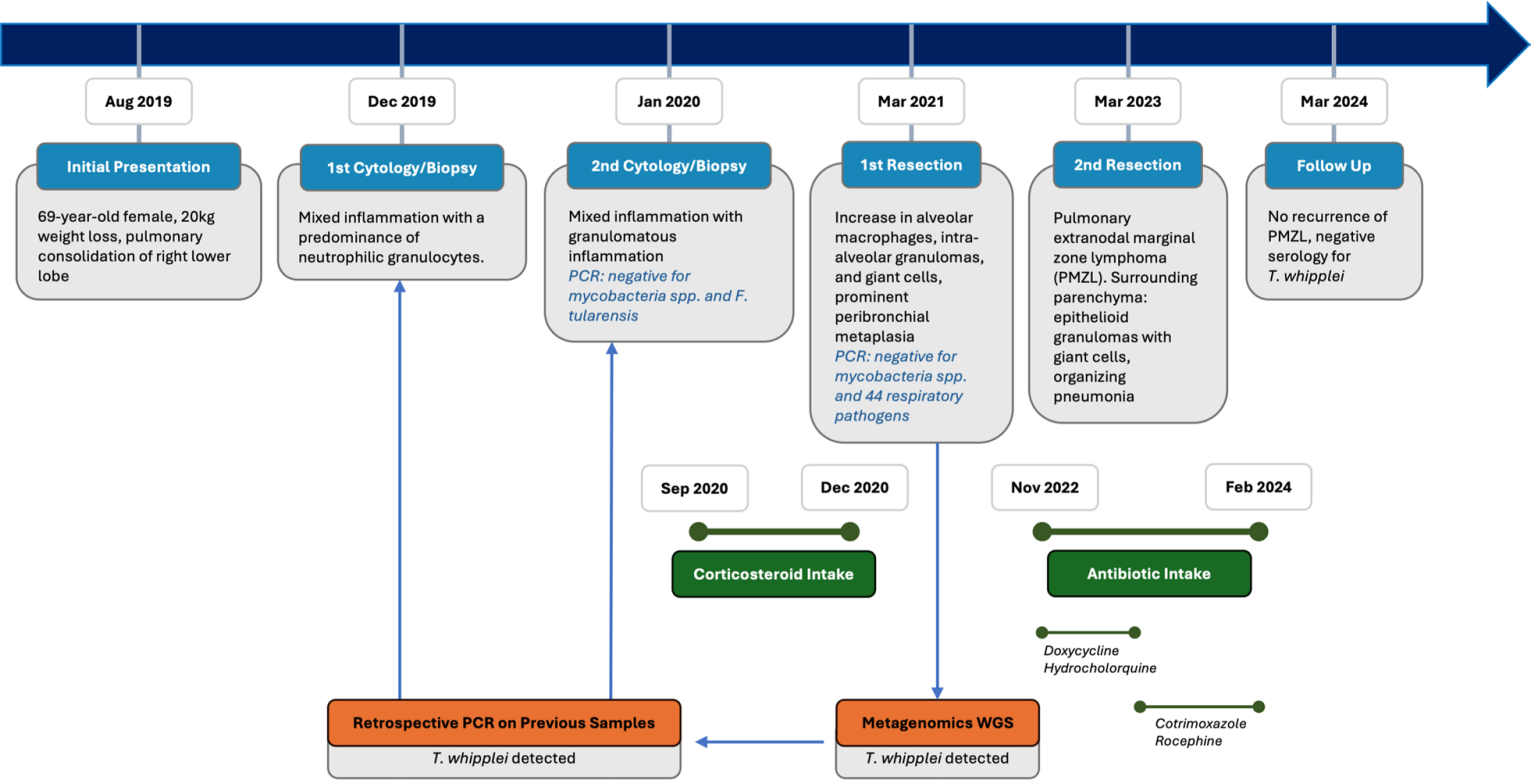
Five-year timeline of clinical events for the index patient with PMZL and detection of *T. whipplei* in various lung tissues using molecular methods (*T. whipplei* specific PCR, mNGS)

The patient first refused antibiotic therapy. In September 2020, a treatment regimen with corticosteroids was initiated (prednisolone 75 mg for 4 weeks; tapering of dosage until December 2020). Despite treatment, the consolidation and symptoms persisted, and the right lower lobe was partially resected (March 2021). Histology revealed an acccumulation of alveolar macrophages, intra-alveolar granulomas with giant cells, and prominent peribronchiolar metaplasia. No intracytoplasmic periodic acid Schiff (PAS) positive granules were detected in alveolar macrophages. Langerhans cell histiocytosis was ruled out by immunohistochemistry (negativity for CD1a). PCR for *Mycobacterium spp*. and 44 other respiratory pathogens in the resection specimen was similarly negative. A mNGS analysis was performed, which detected *T. whipplei*. Subsequent retrospective PCR analyses also detected *T. whipplei* in all previously analysed cytology and biopsy specimens (see Fig. 1 for a full timeline of events). An oesophago-gastro-duodenoscopy was performed, which was unremarkable histologically and yielded negative PCR results for *T. whipplei*. This suggested the lung as the only *T. whipplei* reservoir in this patient. The patient decided against a cerebrospinal fluid analysis, but agreed to antibiotic treatment in November 2022 (initially doxycycline and hydroxychloroquine, later switched to cotrimoxazole and ceftriaxone due to gastrointestinal side effects, Fig. 1).

**Fig. 2:**
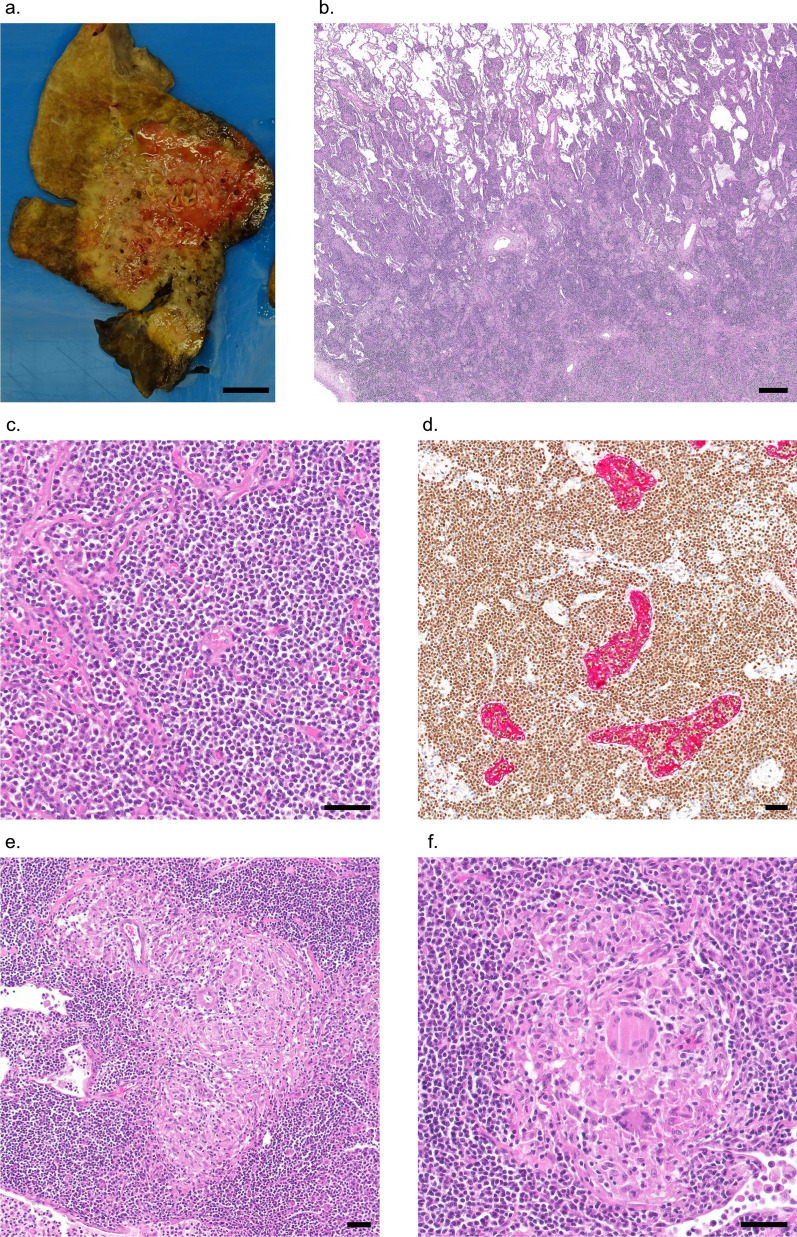
Gross appearance (**a**) and histological findings (**b-f**) of the lung of the index patient. **a** Gross analysis of the lung resection specimen shows an inhomogeneous consolidation of the parenchyma (beige). **b**, **c** Microscopic findings denote a dense lymphocytic infiltrate consisting of monomorphic small lymphocytes (H&E). **d** Double staining for PAX5 (*brown*) and a pan-cytokeratin (AE1/AE3, *red*) demonstrate lymphoepithelial lesions. **e** Numerous intra- and perilesional epithelioid cell granulomas with multinucleated giant cells are similarly observed in adjacent parenchyma (**f**) (H&E). **b** Scale bar: 500 µm. **c–f** Scale bars: 50 µm

The pulmonary consolidation persisted and progressed further, and in March 2023 total lobectomy was performed. Gross analysis of the resection specimen revealed an inhomogeneous consolidation of the lung parenchyma (Fig. 2a). Histological analysis diagnosed a small cell B-cell lymphoma, consistent with PMZL. The surrounding pulmonary parenchyma was remarkable for numerous epithelioid granulomas with giant cell formation as well as perifocal organising pneumonia (Fig. 2b–f). The lymphoma was limited to the lung parenchyma without locoregional spread to perihilar lymph nodes. Of note, a PCR for *T. whipplei* was negative on this resection specimen (4 months after the start of antibiotic therapy).

Eleven days after the operation, the patient presented with a persistent air fistula, which was surgically revised. The patient was dismissed 20 days after admission. She continued antibiotic treatment for 12 months and underwent serological follow-ups every three months. In the last follow-up in March 2024, no peculiarities were reported, and the most recently performed CT and PET scans did not display evidence of a relapse.

### Analysis of a cohort of PMZL and other lymphomas

As we followed the case of this patient longitudinally for several years, we hypothesised that *T. whipplei* may have been a chronic infectious stimulus that promoted the development of PMZL in this patient. Therefore, lung biopsies from a larger cohort of PMZL patients (n = 22) were screened for *T. whipplei* by PCR (Table 1) [21]. In this cohort, one other case with *T. whipplei* positivity in two lung samples, obtained at two different time points, was detected (subsequently referred to as “patient 2”). This result was confirmed by a mNGS analysis.

Patient 2 was a 36-year-old male patient with a migration background. He had been suffering from tuberculosis abroad and sought medical advice in Switzerland for persistent cough (6 months). Imaging showed parenchymal scarring and bilateral pulmonary consolidation suggestive of sarcoidosis, Langerhans cell histiocytosis, or an interstitial lung disease. He was initially treated with broad-spectrum antibiotics (co-amoxicillin and clarithromycin) for 9 days in May 2008. As the consolidations persisted, lung biopsies were taken in July 2008 which showed marked peribronchial and paraseptal lymphoplasmacytic aggregates suspicious for PMZL. Subsequently, the patient developed a pneumothorax and underwent thoracoscopic surgery. Perioperative antibiotic prophylaxis was administered (co-amoxicillin). During the operation, the gross appearance of the lungs (extensive bullae and pleural thickening) warranted two wedge resections. Histological analysis revealed dense infiltrates of atypical small B lymphocytes and evidence of typical lymphoepithelial lesions, consistent with PMZL. Adjacent pulmonary tissue demonstrated granulomatous inflammation with multinucleated giant cells (Supplementary Fig. 1). After the diagnosis, the patient was treated with steroids. This patient had no documented steroid intake prior to thoracoscopic tissue resection. At the point PMZL was diagnosed, *M. tuberculosis* was no longer detected in his lungs (3 samples from distinct anatomical locations were tested by PCR for *M. tuberculosis* and atypical mycobacteria).

Gastric biopsies were taken as part of PMZL staging, which similarly showed infiltrates of MZL. Although *Helicobacter* bacteria were not present, eradication therapy was initiated. *T. whipplei* specific PCRs carried out retrospectively on these gastric biopsies were negative.

For both cases of *T. whipplei* associated PMZL, HTS was performed as previously described (Supplementary Table 2) [21, 25]. The index patient showed a somatic frame-shift insertion mutation in *FOXC1* which was classified as variant of unknown significance (VUS). Patient 2 was found to have a somatic frame-shift insertion mutation in the tumour suppressor gene *EP300*, classified as likely pathogenic. This patient had an additional missense mutation in *SPEN* classified as VUS (Supplementary Table 2). Both mutations of the patient 2 had been found in a previously analysed cohort of PMZL cases from our group [25]. In neither case mutations affecting the nuclear factor Kappa B (NF-κB) or the Notch signalling pathway, both known to be frequently altered in MZL, was detected. The number of mutations detected in both cases of *T. whipplei* associated PMZL was within the range of our previous cohort [21, 25].

**Fig. 3:**
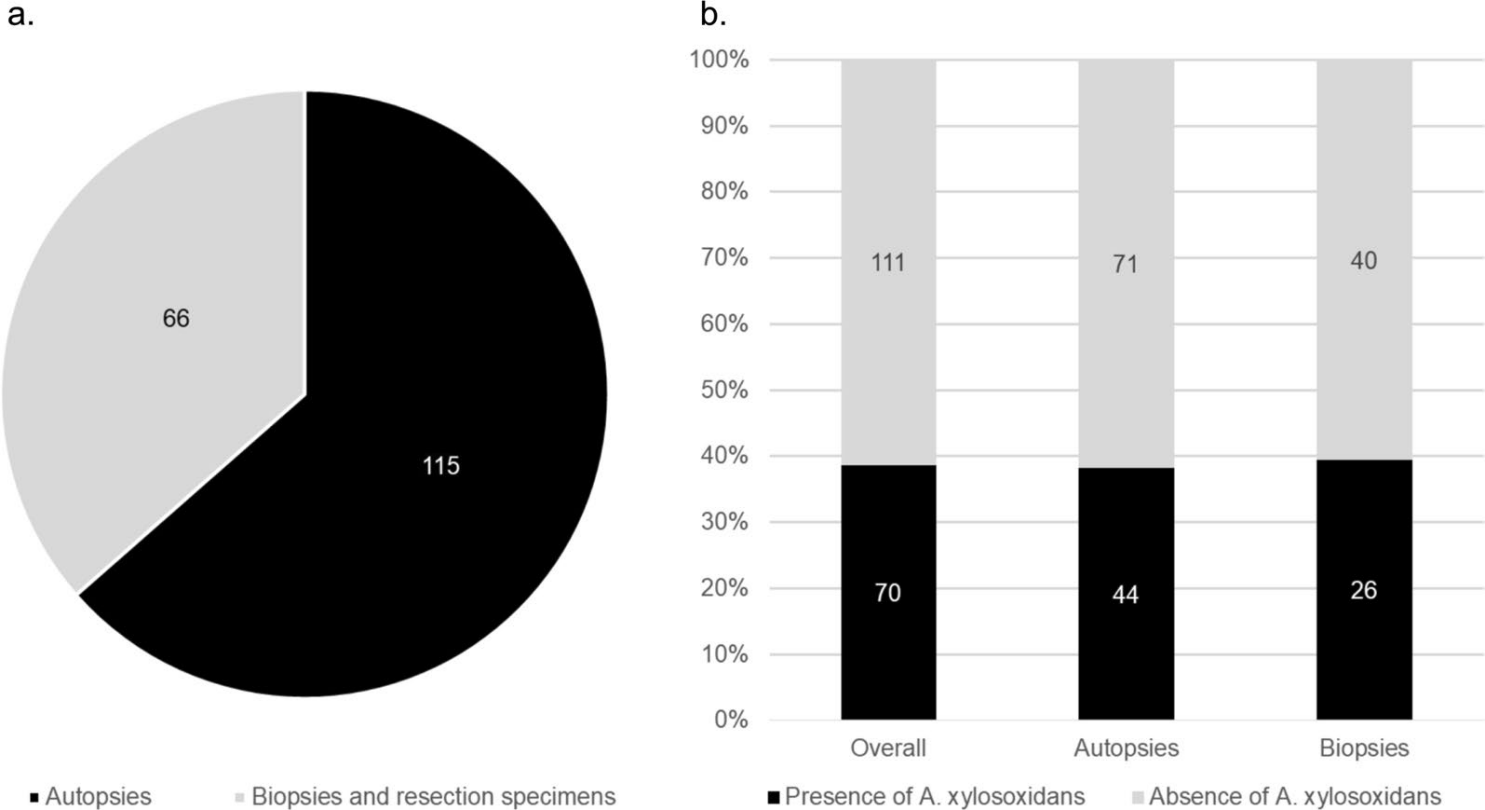
Retrospective analysis of our in-house mNGS dataset **a** consisting of routinely analysed autopsy lungs, lung biopsies and resection specimens (n = 181, of which 115 autopsy lungs and 66 lung biopsies/resections). *T. whipplei* was not detected in any of the previously analysed lung tissues. **b** In contrast, *A. xylosoxidans* was detected in both autopsy and biopsy / resection lung specimens in > 50% of cases, suggesting that it is a common component of the pulmonary microenvironment

All other cases in the PMZL cohort tested negative for *T. whipplei* (Table 1). However, a subset of patients had received broad-spectrum antibiotic treatment regimens for suspected infections within four weeks before undergoing surgery (4/22 cases), and this may have caused false negative PCR results. In addition, all cases with other pulmonary lymphomas (Supplementary Table 3) and normal lung tissue (Supplementary Table 4) were negative for *T. whipplei* by PCR. Similarly, a retrospective analysis of our own metagenomics dataset [22, 26] from routinely analysed lung samples (n = 181, of which 115 autopsy lungs and 66 lung biopsies) for *T. whipplei* yielded negative results. We thus deduce that chronic *T. whipplei* infection or chronic persistence of *T. whipplei* in human lungs is very rare. In contrast, *A. xylosoxidans* bacteria was commonly found in both autopsy and biopsy specimens in > 50% of cases (Fig. 3a, b). This result suggests that *A. xylosoxidans* is part of the normal lung microbiome and unlikely to play a role in the pathogenesis of PMZL.

## Discussion

In this study, we present novel evidence for an association between chronic pulmonary *T. whipplei* infection and PMZL. Pulmonary *T. whipplei* infection may promote neoplastic transformation of B cells, or neoplastic B cell proliferations may provide a niche for *T. whipplei* persistence in the lung. In fact, individual cases have been described in the literature that suggest a pathophysiological connection between *T. whipplei* and B-cell lymphomas, in line with our hypothesis [17–20, 27].

The multifaceted nature of chronic Whipple’s disease is likely due to a combination of factors, both host- and pathogen-dependent, which ultimately result in a failure to clear the bacterium, leading to chronic intracellular oxidative stress and failed antigen presentation. Host-dependent factors include an increased likelihood to develop chronic Whipple’s disease or persistence in patients with HLA-B27 positivity [28], suggesting an impairment of cell-mediated immunity [17]. Macrophage dysfunction has been postulated to be a hallmark of chronic Whipple’s disease, for example due to a decrease of interleukin-12 (IL-12), disturbed phagocytosis and antigen presentation. This, in turn, decreases interferon gamma (IFNγ), dysregulates NK-, T- and B-cell response [14, 29, 30], and ultimately contributes to lymphomagenesis [10]. Interestingly, clinical evidence suggests that patients with a history of immunosuppression or receipt of immunosuppressive agents, especially TNFα inhibitors, were more likely to develop systemic Whipple’s disease with rapid deterioration directly or soon after medication onset [31–33]. Although none of the patients tested positive for *T. whipplei* in our cohort has a known prior history of immunosuppression, it should be considered as one of the potential risk factors for systemic disease.

Another important factor in the pathogenesis of chronic Whipple’s disease is NF-κB-mediated signalling, downstream of bacterial sensors such as Toll-like receptor 4 (TLR4) [34]. Previous studies suggest that bacterial infections may have activating effects on the B-cell receptor (BCR) and the NF-κB pathway in B cells, further supporting the pathophysiological role of chronic antigen stimulation in lymphomagenesis [35]. It has been shown that NF-κB-mediated oxidative stress can induce de novo genomic rearrangements, as was previously shown for the *TMPRSS2-ERG* gene fusion in prostate cancer [36]. The role of NF-κB in PMZL lymphomagenesis is also supported by HTS analyses [21], which identified mutations and translocations of *TNFAIP3, BCL10, CARD11, MYD88, TNFRSF14* and *MALT1* in almost 60% of PMZL. All of these alterations have been shown to influence the activity of the NF-kB pathway [37, 38]. Interestingly, both cases of *T. whipplei*-associated PMZL showed comparatively few mutations and, in particular, no mutations in genes related to the NF-κB or the Notch signalling pathway and no mutations in chromatin modifier encoding genes characteristic for PMZL [21, 25]. A similar pattern is observed in diffuse large B-cell lymphoma (DLBCL), where the mutational profiles and load differ significantly between EBV-positive and negative cases [39, 40]. These findings suggest that *T. whipplei* infection may compensate for the missing mutations in these PMZL, thus promoting lymphomagenesis.

Our data on the association of *T. whipplei* and PMZL suggests a potential causative link between *T. whipplei* infection and PMZL requiring further confirmative studies. MZL in other localisations are similarly associated with various chronic infections [5–7], and patients with PMZL have been successfully treated with prolonged clarithromycin therapy, further corroborating a potential infectious cause of this B-cell lymphoma [41]. However, several uncertainties remain. Patient 2 had a history of *M. tuberculosis*, which might have contributed to the development of PMZL through chronic inflammatory stimulation. Only a minority of pathogens are known to chronically persist in the lung, which is a fundamental prerequisite for chronic inflammatory stimulation. Indeed, *M. tuberculosis* DNA has been found by *in-situ* PCR within lungs of individuals who have died from causes other than tuberculosis [42, 43]. A previously reported case suggested a temporal link between the occurrence of PMZL and pulmonary mycobacterial infection and regression of PMZL after antibiotic treatment for concomitant pulmonary tuberculosis [44]. Based on this data, we cannot exclude a pathophysiological connection between chronic mycobacterial infection and the occurrence of PMZL in patient 2. However, a PCR on the lung tissue taken at the time of PMZL diagnosis was negative for mycobacteria (*M. tuberculosis* and atypical mycobacteria).

A history of perioperative broad-spectrum antibiotic treatment in 4 of 22 cases might have influenced the negative PCR results of the cohort, although the rate of tissue penetration of differing antibiotic regimens should not be overestimated. In general, antibiotic intake can cause false negative results in PCR analyses, as demonstrated in the second lung resection specimen of the index patient, which turned out negative *T. whipplei* after four months of antibiotic treatment (Fig. 1). The elimination of causative bacterial species by means of antibiotic therapy could eventually become a component of PMZL treatment, similar to *Helicobacter* eradication in gastric MZL [45–48]. As other bacterial infections could be potential PMZL drivers, it will be important to study larger PMZL cohorts with a non-targeted approach such as mNGS, rather than by means of species-specific PCR only.

Non-targeted analyses conducted so far have shown no evidence of any causative link between any pathogen and PMZL and, in particular, have refuted the suggested role of A. xylosoxidans [10]. Our data supports *A. xylosoxidans* as a component of the normal lung microbiome and do not suggest it as a potential contaminant. Firstly, its pattern of detection is neither systematic nor isolated. In contrast, our dataset is systematically contaminated by *Sphingomonas spp.* (plant-related bacteria) and occasionally displays abundant contamination by *Methylobacterium spp.*, which are known to contaminate DNA extraction reagents [49]. In contrast, *A. xylosoxidans* is evenly represented in our cohort. Secondly, we have recently implemented improvements to our bioinformatics pipeline based on the latest developments and recommendations in the field of metagenomics [50, 51]. We now perform a second human read depletion using the latest and complete version of the human genome (CHM13). This additional filtering has enabled us to identify and remove contaminants upon reanalysis of our data. *A. xylosoxidans* was not identified as a bioinformatics artefact during this process, further suggesting its actual presence in our samples.

## Conclusions

Our data highlights a potential role of *T. whipplei* infection in pulmonary granulomatous lung disease with an unclear aetiology and establishes a potential association between this bacterium and PMZL. In addition, our study emphasises the utility of metagenomics in daily pathology practice, which could significantly impact on the treatment regimen of PMZL. This introduces the possibility of first-line antibiotic therapy in cases of PMZL where *T. whipplei* is detected. Future studies featuring larger cohorts of patients with unclear granulomatous lung disease and/or PMZL screened for *T. whipplei* are required to further strengthen the hypothesis that *T. whipplei* may be a potential driver of PMZL.

### Supplementary Information

Additional file 1.

Additional file 2.

## Data Availability

The HTS data acquired for this study is accessible via the European Nucleotide Archive (ENA, https://www.ebi.ac.uk/) under the accession no. PRJEB73816.

## Abbreviations

BAL: Bronchoalveolar lavage
BCR: B cell receptor
CT: Computed tomography
DLBCL: Diffuse large B-cell lymphoma
EBV: Epstein-Barr virus
EDTA: Ethylenediaminetetraacetic acid
FFPE: Formalin-fixed paraffin-embedded
HIV: Human immunodeficiency virus
HLA: Human leukocyte antigen
HPV: Human papilloma virus
HTS: High-throughput sequencing
IFNγ: Interferon gamma
IL: Interleukin
KMT2D: Histone-lysine N-methyltransferase 2D
MALT: Mucosa-associated lymphatic tissue
MZL: Marginal zone lymphoma
NF-kB: Nuclear factor Kappa B
mNGS: Metagenomic next-generation sequencing
NK: Natural killer
PAS: Periodic acid Schiff
PCR: Polymerase chain reaction
PET: Positron emission tomography
PMZL: Pulmonary marginal zone lymphoma
rRNA: Ribosomal ribonucleic acid
spp.: Species
TGFβ: Transforming growth factor Beta
TLR4: Toll-like receptor 4
TMPRSS2-ERG: Transmembrane serine protease 2/ETS transcription factor
TNFAIP3: Tumour necrosis factor, alpha induced protein 3
VUS: Variant of unknown significance

## References

[1] De Martel C, Georges D, Bray F, Ferlay J, Clifford GM. Global burden of cancer attributable to infections in 2018: a worldwide incidence analysis. Lancet Glob Health. 2020;8:e180–90. 31862245 10.1016/S2214-109X(19)30488-7

[2] Swerdlow SH, Campo E, Pileri SA, Harris NL, Stein H, Siebert R, et al. The 2016 revision of the World Health Organization classification of lymphoid neoplasms. Blood. 2016;127:2375–90. 26980727 10.1182/blood-2016-01-643569 PMC4874220

[3] Troppan K, Wenzl K, Neumeister P, Deutsch A. Molecular Pathogenesis of MALT Lymphoma. Gastroenterol Res Pract. 2015;2015:1–10. 10.1155/2015/102656 PMC4397421 25922601

[4] Rossi D, Bertoni F, Zucca E. Marginal-Zone Lymphomas. N Engl J Med. 2022;386:568–81. 35139275 10.1056/NEJMra2102568

[5] Hu Q, Zhang Y, Zhang X, Fu K. Gastric mucosa-associated lymphoid tissue lymphoma and Helicobacter pylori infection: a review of current diagnosis and management. Biomark Res. 2016;4:15. 27468353 10.1186/s40364-016-0068-1 PMC4962427

[6] Köller MC, Aigelsreiter A. Chlamydia psittaci in ocular adnexal MALT lymphoma: a possible causative agent in the pathogenesis of this disease. Curr Clin Micro Rpt. 2018;5:261–7. 10.1007/s40588-018-0108-8

[7] Travaglino A, Varricchio S, Pace M, Russo D, Picardi M, Baldo A, et al. *Borrelia burgdorferi* in primary cutaneous lymphomas: a systematic review and meta-analysis. J Deutsche Derma Gesell. 2020;18:1379–84. 10.1111/ddg.14289 33029842

[8] Suarez F, Lortholary O, Hermine O, Lecuit M. Infection-associated lymphomas derived from marginal zone B cells: a model of antigen-driven lymphoproliferation. Blood. 2006;107:3034–44. 16397126 10.1182/blood-2005-09-3679

[9] Adam P, Czapiewski P, Colak S, Kosmidis P, Tousseyn T, Sagaert X, et al. Prevalence of Achromobacter xylosoxidans in pulmonary mucosa-associated lymphoid tissue lymphoma in different regions of Europe. Br J Haematol. 2014;164:804–10. 24372375 10.1111/bjh.12703

[10] Borie R, Wislez M, Antoine M, Copie-Bergman C, Thieblemont C, Cadranel J. Pulmonary mucosa-associated lymphoid tissue lymphoma revisited. Eur Respir J. 2016;47:1244–60. 26797028 10.1183/13993003.01701-2015

[11] Fenollar F, Lagier J-C, Raoult D. Tropheryma whipplei and Whipple’s disease. J Infect. 2014;69:103–12. 24877762 10.1016/j.jinf.2014.05.008

[12] Martinetti M, Biagi F, Badulli C, Feurle GE, Müller C, Moos V, et al. The HLA Alleles DRB1*13 and DQB1*06 Are Associated to Whipple’s Disease. Gastroenterology. 2009;136:2289–94. 19208355 10.1053/j.gastro.2009.01.051

[13] Schöniger-Hekele M, Petermann D, Weber B, Müller C. Tropheryma whipplei in the environment: survey of sewage plant influxes and sewage plant workers. Appl Environ Microbiol. 2007;73:2033–5. 17277223 10.1128/AEM.02335-06 PMC1828826

[14] Dolmans RAV, Boel CHE, Lacle MM, Kusters JG. Clinical manifestations, treatment, and diagnosis of tropheryma whipplei infections. Clin Microbiol Rev. 2017;30:529–55. 28298472 10.1128/CMR.00033-16 PMC5355640

[15] Ruffer N, Holzer M-T, Gkanatsas Y, Schinglerová I, Boro D, Krusche M, et al. Chronische tropheryma-whipplei-infektion: eine wichtige Differentialdiagnose der therapierefraktären Polyarthritis. Z Rheumatol. 2023;82:885–91. 35384513 10.1007/s00393-022-01194-5 PMC10695860

[16] Jutant E-M, Roche A, Boucly A, Camboulive A, Jevnikar M, Jais X, et al. Whipple’s disease: a rare and life-threatening cause of pulmonary hypertension. Pulmonary hypertension [Internet]. European Respiratory Society; 2021 [cited 2023 Dec 29]. p. PA3599. Available from: http://erj.ersjournals.com/lookup/doi/10.1183/13993003.congress-2021.PA3599

[17] Gillen CD, Coddington R, Monteith PG, Taylor RH. Extraintestinal lymphoma in association with Whipple’s disease. Gut. 1993;34:1627–9. 7694890 10.1136/gut.34.11.1627 PMC1374435

[18] Gruner U, Goesch P, Donner A, Peters U. Morbus Whipple und Non-Hodgkin-Lymphom. Z Gastroenterol. 2001;39:305–9. 11367979 10.1055/s-2001-12865

[19] Cadenas F, Sánchez-Lombraña JL, Pérez R, Lomo FJ, Madrigal Rubiales B, Vivas S, et al. Persistent leucocytosis as initial manifestation of Whipple’s disease and development of gastric cancer in the follow up. Rev Esp Enferm Dig. 1999;91:785–8. 10601772

[20] Wang S, Ernst LM, Smith BR, Tallini G, Howe JG, Crouch J, et al. Systemic Tropheryma whippleii infection associated with monoclonal B-cell proliferation: a Helicobacter pylori-type pathogenesis? Arch Pathol Lab Med. 2003;127:1619–22. 14632565 10.5858/2003-127-1619-STWIAW

[21] Vela V, Juskevicius D, Dirnhofer S, Menter T, Tzankov A. Mutational landscape of marginal zone B-cell lymphomas of various origin: organotypic alterations and diagnostic potential for assignment of organ origin. Virchows Arch. 2022;480:403–13. 34494161 10.1007/s00428-021-03186-3 PMC8986713

[22] Nienhold R, Mensah N, Frank A, Graber A, Koike J, Schwab N, et al. Unbiased screen for pathogens in human paraffin-embedded tissue samples by whole genome sequencing and metagenomics. Frontiers in Cellular and Infection Microbiology [Internet]. 2022 [cited 2024 Jan 6];12. Available from: https://www.frontiersin.org/articles/10.3389/fcimb.2022.968135 10.3389/fcimb.2022.968135 PMC9530700 36204644

[23] Talwalkar SS, Valbuena JR, Abruzzo LV, Admirand JH, Konoplev SN, Bueso-Ramos CE, et al. MALT1 gene rearrangements and NF-kappaB activation involving p65 and p50 are absent or rare in primary MALT lymphomas of the breast. Mod Pathol. 2006;19:1402–8. 16917511 10.1038/modpathol.3800668

[24] Ivanova V-S, Davies J, Menter T, Wild D, Müller A, Krasniqi F, et al. Primary bone diffuse large B-cell lymphoma (PB-DLBCL): a distinct extranodal lymphoma of germinal centre origin, with a common EZB-like mutational profile and good prognosis. Histopathology. 2024;84:525–38. 37965677 10.1111/his.15096

[25] Vela V, Juskevicius D, Prince SS, Cathomas G, Dertinger S, Diebold J, et al. Deciphering the genetic landscape of pulmonary lymphomas. Mod Pathol. 2021;34:371–9. 32855441 10.1038/s41379-020-00660-2

[26] Nienhold R, Ciani Y, Koelzer VH, Tzankov A, Haslbauer JD, Menter T, et al. Two distinct immunopathological profiles in autopsy lungs of COVID-19. Nat Commun. 2020;11:5086. 33033248 10.1038/s41467-020-18854-2 PMC7546638

[27] Sauter C, Kurrer MO. Intracellular bacteria in Hodgkin’s disease and sclerosing mediastinal B-cell lymphoma: Sign of a bacterial etiology? Swiss Med Wkly. 2002;132:312–5. 12362281 10.4414/smw.2002.09976

[28] Tison A, Preuss P, Leleu C, Robin F, Le Pluart A, Vix J, et al. Rheumatological features of Whipple disease. Sci Rep. 2021;11:12278. 34112875 10.1038/s41598-021-91671-9 PMC8192552

[29] Crayne CB, Albeituni S, Nichols KE, Cron RQ. The Immunology of Macrophage Activation Syndrome. Frontiers in Immunology [Internet]. 2019 [cited 2023 Dec 26];10. Available from: https://www.frontiersin.org/articles/10.3389/fimmu.2019.00119 10.3389/fimmu.2019.00119 PMC6367262 30774631

[30] Desnues B, Ihrig M, Raoult D, Mege J-L. Whipple’s disease: a macrophage disease. Clin Vaccine Immunol. 2006;13:170–8. 16467322 10.1128/CVI.13.2.170-178.2006 PMC1391942

[31] Lagier J-C, Lepidi H, Raoult D, Fenollar F. Systemic Tropheryma whipplei: clinical presentation of 142 patients with infections diagnosed or confirmed in a reference center. Medicine (Baltimore). 2010;89:337–45. 20827111 10.1097/MD.0b013e3181f204a8

[32] Marth T. Tropheryma whipplei, Immunosuppression and whipple’s disease: from a low-pathogenic, environmental infectious organism to a rare. Multifaceted Inflamm Complex Dig Dis. 2015;33:190–9. 10.1159/000369538 25925922

[33] Marth T. Complicated Whipple’s disease and endocarditis following tumor necrosis factor inhibitors. World J Cardiol. 2014;6:1278–84. 25548618 10.4330/wjc.v6.i12.1278 PMC4278163

[34] Liu J, Xiang J, Li X, Blankson S, Zhao S, Cai J, et al. NF-κB activation is critical for bacterial lipoprotein tolerance-enhanced bactericidal activity in macrophages during microbial infection. Sci Rep. 2017;7:40418. 28079153 10.1038/srep40418 PMC5227741

[35] Thurner L, Hartmann S, Fadle N, Regitz E, Kemele M, Kim Y-J, et al. Lymphocyte predominant cells detect Moraxella catarrhalis-derived antigens in nodular lymphocyte-predominant Hodgkin lymphoma. Nat Commun. 2020;11:2465. 32424289 10.1038/s41467-020-16375-6 PMC7235000

[36] Mani RS, Amin MA, Li X, Kalyana-Sundaram S, Veeneman BA, Wang L, et al. Inflammation-induced oxidative stress mediates gene fusion formation in prostate cancer. Cell Rep. 2016;17:2620–31. 27926866 10.1016/j.celrep.2016.11.019 PMC5147555

[37] Serramito-Gómez I, Boada-Romero E, Slowicka K, Vereecke L, Van Loo G, Pimentel-Muiños FX. The anti-inflammatory protein TNFAIP3/A20 binds the WD40 domain of ATG16L1 to control the autophagic response, NFKB/NF-κB activation and intestinal homeostasis. Autophagy. 2019;15:1657–9. 31184523 10.1080/15548627.2019.1628549 PMC6693451

[38] Ortega-Molina A, Boss IW, Canela A, Pan H, Jiang Y, Zhao C, et al. The histone lysine methyltransferase KMT2D sustains a gene expression program that represses B cell lymphoma development. Nat Med. 2015;21:1199–208. 26366710 10.1038/nm.3943 PMC4676270

[39] Menter T, Juskevicius D, Alikian M, Steiger J, Dirnhofer S, Tzankov A, et al. Mutational landscape of B-cell post-transplant lymphoproliferative disorders. Br J Haematol. 2017;178:48–56. 28419429 10.1111/bjh.14633

[40] Zhou Y, Xu Z, Lin W, Duan Y, Lu C, Liu W, et al. Comprehensive genomic profiling of EBV-positive diffuse large b-cell lymphoma and the expression and clinicopathological correlations of some related genes. Front Oncol. 2019;9:683. 31403034 10.3389/fonc.2019.00683 PMC6669985

[41] Ishimatsu Y, Mukae H, Matsumoto K, Harada T, Hara A, Hara S, et al. Two cases with pulmonary mucosa-associated lymphoid tissue lymphoma successfully treated with clarithromycin. Chest. 2010;138:730–3. 20822996 10.1378/chest.09-2358

[42] Hernández-Pando R, Jeyanathan M, Mengistu G, Aguilar D, Orozco H, Harboe M, et al. Persistence of DNA from Mycobacterium tuberculosis in superficially normal lung tissue during latent infection. Lancet. 2000;356:2133–8. 11191539 10.1016/S0140-6736(00)03493-0

[43] Inadome Y, Ikezawa T, Oyasu R, Noguchi M. Malignant lymphoma of bronchus-associated lymphoid tissue (BALT) coexistent with pulmonary tuberculosis. Pathol Int. 2001;51:807–11. 11881735 10.1046/j.1440-1827.2001.01272.x

[44] Gaur S, Trayner E, Aish L, Weinstein R. Bronchus-associated lymphoid tissue lymphoma arising in a patient with bronchiectasis and chronic Mycobacterium avium infection. Am J Hematol. 2004;77:22–5. 15307101 10.1002/ajh.20136

[45] Parsonnet J, Hansen S, Rodriguez L, Gelb AB, Warnke RA, Jellum E, Orentreich N, Vogelman JH, Friedman GD. Helicobacter pylori Infection and Gastric Lymphoma. N Engl J Med. 1994;330(18):1267–71. 8145781 10.1056/NEJM199405053301803

[46] Raderer M, Wöhrer S, Kiesewetter B, Dolak W, Lagler H, Wotherspoon A, et al. Antibiotic treatment as sole management of Helicobacter pylori-negative gastric MALT lymphoma: a single center experience with prolonged follow-up. Ann Hematol. 2015;94:969–73. 25579756 10.1007/s00277-014-2298-3

[47] Wotherspoon AC, Doglioni C, Diss TC, Pan L, Moschini A, de Boni M, et al. Regression of primary low-grade B-cell gastric lymphoma of mucosa-associated lymphoid tissue type after eradication of Helicobacter pylori. Lancet. 1993;342:575–7. 8102719 10.1016/0140-6736(93)91409-F

[48] Park JB, Koo JS. Helicobacter pylori infection in gastric mucosa-associated lymphoid tissue lymphoma. World J Gastroenterol. 2014;20:2751–9. 24659867 10.3748/wjg.v20.i11.2751 PMC3961970

[49] Salter SJ, Cox MJ, Turek EM, Calus ST, Cookson WO, Moffatt MF, et al. Reagent and laboratory contamination can critically impact sequence-based microbiome analyses. BMC Biol. 2014;12:87. 25387460 10.1186/s12915-014-0087-z PMC4228153

[50] Simner PJ, Salzberg SL. The human “contaminome” and understanding infectious disease. N Engl J Med. 2022;387:943–6. 36069879 10.1056/NEJMcibr2208625

[51] Gihawi A, Ge Y, Lu J, Puiu D, Xu A, Cooper CS, et al. Major data analysis errors invalidate cancer microbiome findings. MBio. 2023;14:e0160723. 37811944 10.1128/mbio.01607-23 PMC10653788

