# Supplementary figures and images for: Two cases demonstrate an association between Tropheryma whipplei and pulmonary marginal zone lymphoma
Source: Infect Agent Cancer. 2024 Jul 27;19:33. doi: 10.1186/s13027-024-00597-0 (PMC11282790; doi:10.1186/s13027-024-00597-0)

Supplementary Figure 1

a.

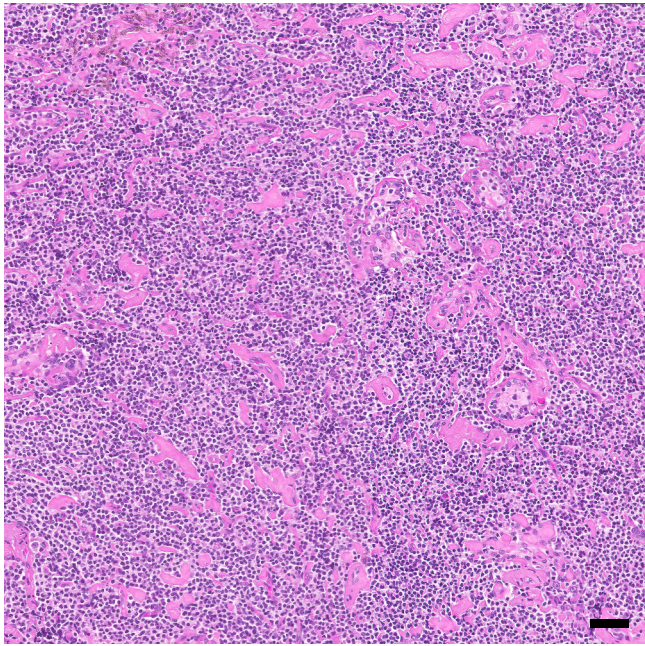

b.

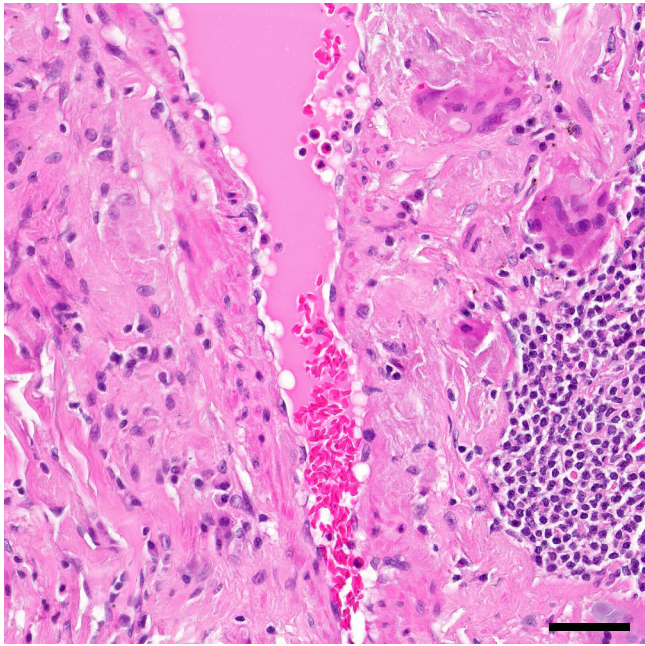

Supplement: Supplementary file 2 — Additional file 2. [file 13027_2024_597_MOESM2_ESM.pdf]
